# Voice symptoms in teachers during distance teaching: a survey during the COVID-19 pandemic in Finland

**DOI:** 10.1007/s00405-021-06960-w

**Published:** 2021-07-04

**Authors:** M. Patjas, H. Vertanen-Greis, P. Pietarinen, A. Geneid

**Affiliations:** 1 Department of Otorhinolaryngology and Phoniatrics - Head and Neck Surgery, University of Helsinki and Helsinki University Hospital, Helsinki, Finland; 2 Turku Clinical Research Centre, University of Turku, Turku, Finland

**Keywords:** Voice, Teacher, Voice symptoms, Distance learning, Covid-19, Background noise, Air quality, Work ability

## Abstract

**Purpose:**

Due to the coronavirus disease of 2019 (COVID-19), teachers during the pandemic have had to adapt to online teaching at short notice. This study aims to investigate the voice symptoms and their environmental risk factors as well as the work ability associated with distance teaching and to compare these with symptoms in previous contact teaching.

**Methods:**

We conducted a survey of 121 primary and secondary school teachers across Finland. The survey was advertised online through social media and the replies collected from voluntarily participating teachers.

**Results:**

During distance teaching vocal symptoms appeared less often than in school with 71% teachers experiencing them in regular teaching and 44% in distance teaching, VHI result decreased from 7.88 in school teaching to 4.58 in distance teaching. Acoustic conditions were reported to be more suitable in distance teaching with 73% of teachers finding them adequate during distance teaching in comparison to 46% for those in regular teaching. Background noise was the most disturbing factor for a teacher’s voice in the classroom and in distance teaching and this was even more conspicuous in the classroom. Also, subjectively experienced poor indoor air quality at school influenced the voice negatively. Further, voice problems were associated with increased subjective stress levels and reduced ability to work.

**Conclusion:**

Distance teaching has affected teachers’ voices in a positive way compared with regular teaching. This difference is likely to be due to better acoustics and indoor air quality in distance teaching conditions.

**Supplementary Information:**

The online version contains supplementary material available at 10.1007/s00405-021-06960-w.

## Introduction

A new coronavirus (SARS-CoV-2) has caused a global pandemic which began in spring 2020 of the northern hemisphere. In response to the pandemic, the Finnish government ordered basic education and general upper secondary education to be delivered as distance teaching, commencing on 18 March 2020 (Finnish Government decree, 2020 #36). Schools were permitted to choose platforms that are appropriate for distance teaching, with teachers working mainly from their homes. This regulation lasted for eight weeks, ending on 13 May 2020. According to a recent survey by the Trade Union of Education in Finland (OAJ) [1], the transformation from contact teaching in Finnish schools to distance teaching posed challenges to the content of teaching, the use of technological resources and teachers’ interaction with pupils. General distance teaching practices were not applied to teaching younger children. The new teaching conditions caused concern among teachers, with one-fifth rating their well-being at work as poor.

The voice is an essential tool in the teaching profession, and voice problems are more common among teachers than those in other occupations [2]. According to a recent study, half of the Finnish teachers suffer from voice disorders [3], and voice problems are also found to be significantly associated with work ability of teachers in Finland, Sweden and other countries [4–6].

Vocal load can be defined as “vocal doses”, referring to the amount of vocal fold tissue exposure to vibration during phonation [7]. Hunter et al. [8] presented a modelled definition of the terms “vocal fatigue”, “vocal effort”, “vocal load” and “vocal loading” and proposed the use of two new terms “vocal demand” and “vocal demand responses”, standardised definitions should be redefined in future work. However, teachers generally experience increased vocal load in their daily work [7, 9]. This profession not only requires prolonged voice use but also involves other loading factors, including background noise, long speaking distance, poor room acoustics and lack of adequate technical acoustic equipment [10, 11]. Thus, it is obvious that a marked vocal load is present in teaching work, including distance teaching. In occupations with a high voice demand and potentially increased vocal load, the aetiology of vocal symptoms may be more environmental [12]. Distance teaching entails a change in the teaching environment, with teaching occurring from the home. This may involve a change in acoustics, length of teaching time and background noise. Vocal performance in the classroom differs considerably from distance teaching, with the teacher being alone in a room at home and having contact with students only via the internet. In addition, other indoor air problems that are linked to voice symptoms, such as ventilation, change substantially during distance teaching. Ventilation problems are a common concern in Finnish schools and may even multiply the risks of voice symptoms [13, 14]. In addition, organic and chemical impurities trigger allergic or inflammatory reactions in the larynx and exposure to organic dust may cause vocal symptoms. Humidity and temperature also affect the indoor air quality [14].

Despite the importance of the voice in a teacher’s work ability, to date, no attention has been paid to voice symptoms associated with distance teaching. Previous studies have suggested that voice problems, stress and also insufficient indoor environment are associated with the ability to work, with the absence of sickness being an indicator of it [6]. Our aim here was to determine whether the distance teaching arrangements along with the changed work environment are associated with teachers’ voice symptoms. Based on such presumed changes, we hypothesised that a teacher’s voice would improve, whereas their work ability would decrease during the period of distance teaching because of vast changes in the working environment. We anticipated that classroom teaching would challenge the voice more than distance teaching due to commonly encountered stressors such as poor acoustics, high levels of background noise and increased vocal effort to speak loudly enough to be heard by all students. On the other hand, among other things, the communication expressiveness of teaching professionals depends on non-verbal communication [15] to enable good interaction with students [16]. Therefore, it is possible that teaching without any direct contact with students may force teachers to compensate for the reduced body language by vocal nuances.

The purpose of this study was to understand the effect of acoustic conditions, background noise, indoor air quality, work ergonomics and technical challenges on teachers’ voices, as well as to compare subjective negative stress levels and work ability during distance teaching in comparison to regular classroom teaching. In this regard, the time before distance teaching served as the baseline for such a comparison between the two periods.

## Subjects and methods

We undertook a survey among basic and upper secondary school teachers across Finland in May 2020 at the end of the first wave of the pandemic (Supplementary Appendices S1 and S2). The survey was advertised through a link shared on social media with support from the Trade Union of Education in Finland (OAJ), which announced the study to its members. However, considering that the link depends on a teacher being a member of a specific social media channel as well as her/his online status and interest in participating in the survey, estimating a dropout rate is not possible. All class and subject teachers working in Finnish-speaking basic and secondary schools were included in the study, with exception of teachers of basic grades 1–3 and special education teachers. The forementioned groups were excluded since they continued partially with regular teaching and were exempted partially or totally from the government directive on distance teaching. Their exclusion from this study was done to ensure a homogeneous sample fulfilling the requirement of 100% distance teaching during the study period. Thus, the age range of the pupils was 10 to 18 years.

### Survey

The survey included variables assessed by the teachers in terms of their severity before and during the distance teaching period that lasted for eight weeks in April and May 2020. Survey collection was started at the end of May 2020 and lasted for six weeks. Teachers had recent valid memory of the situation before distance teaching and differences between the two period were freshly experienced.

Such variables included the Voice Handicap Index (VHI-10) [17, 18], frequency of voice problems, stress level [19] at work and in private life, health, work ability and environmental circumstances such as noise, indoor air quality and audio-visual techniques used.

The VHI-10 was used to evaluate the vocal symptoms in our survey. The original Voice Handicap Index (VHI) was developed with 30 questions [17] using a diverse sample of patients with voice disorders, representing the breadth of pathology in most clinical settings. This was intentional targeting to create a scale that could be generalised to other clinics and would have widespread application. The VHI has several potential uses in the clinical practice of speech-language pathology. Later, an abbreviated voice handicap assessment instrument (VHI-10) was developed by reducing the number of questions from 30 to 10. It takes less time for the patient to complete without loss of validity, and it can replace the VHI as an instrument to quantify patients' perception of their voice handicap [18].

Further, we enquired about the frequency of vocal problems (daily, weekly, less frequently, not at all) as well as the average duration of daily speaking.

We also enquired about symptoms of stress. Theoretically stress symptoms can be classified into positive and negative ones. Positive stress is conceptualised as the perception of being in control of a situation, and the negative one as the perception of lacking control over a situation. [20] We assessed what is regarded as negative stress by focusing on some of its symptoms (tension, restlessness, nervousness, anxiety, difficulty in falling asleep). The question was modified from a validated single-item question [21]. It was recorded on a five-point Likert scale as “no stress”, “only a little”, “some”, “rather much” and “very much".

Moreover, we asked about acoustic circumstances and if they were more pleasant at home or at school. It is obvious that these situations are different for each participant, but luckily the differences are not highly distinguishable in Finland.

The other variables were assessed according to the general situation. We assessed work ability by using the Work Ability Score (WAS) [22]. A validated single-item question on current work ability was recorded on a scale from 0 (“completely unable to work”) to 10 (“work ability at its best”). WAS is a part of the Work Ability Index, which has the highest discriminating power of the entire index. In the analysis, we used the classification of WAS found to correspond best with that of the Work Ability Index [23]: poor (0–5 points), moderate (6–7), good (8–9), and excellent (10). We combined good and excellent work ability, and thus, used three categories in the analyses. We then enquired about workload during distance learning relative to contact teaching with the options less, slightly less, the same, slightly more and more.

We also asked about background noise, technical challenges, subjectively perceived poor indoor air quality and poor working ergonomics (e.g., worktop) in terms of their impact on regular or distance teaching. Furthermore, we posed questions about the use of audio equipment (headphones, separate microphone, headset or conference speaker) during distance learning and audio amplifiers in the classroom.

Background variables included sex, age (grouped into 18–29, 30–39, 40–49, 50–59, 60–68 years) and region of employment. Profession was classified into two categories: class teacher and subject teacher. In addition, we enquired about education level attained using three categories: primary, secondary and upper secondary education. The main teaching subject was ascertained, as was the number of working years. Regarding voice-related diseases, we asked whether the subject had a chronic pulmonary disease (e.g., asthma, chronic obstructive pulmonary disease), allergic rhinitis or reflux disease and if any operations on the larynx or hypopharynx had been performed. Smoking was assessed using three categories: never smoked, ex-smoker, current smoker; the latter two options were combined for the analysis.

### Data analysis

Pearson’s Chi-squared test and Fisher’s exact test were used to examine the responses to questions on the frequency of vocal symptoms before and during distance learning. We used the Mann–Whitney U-test to compare the results of VHI-10, voice symptoms, work ability and the dichotomous variables. For ordinal and continuous variables, we applied the Spearman correlation. The analysis was performed using IBM SPSS Statistics, version 26.

### Ethics approval

The Ethics Board of Helsinki University Hospital approved the study protocol (Statement 1580/2020). Participants enrolled online voluntarily, giving their written informed consent to receive e-authorisation, which is linked to the participant’s social security number; this allowed us to exclude any participation by other persons, to avoid duplicate responses and to combine the results with future follow-up findings. The collected data were processed in accordance with the General Data Protection Regulation (GDPR), and after the inquiry closure, the data were immediately pseudonymised.

## Results

Altogether 121 teachers participated in the study with 52% participating from Uusimaa region that has over 30% of the Finnish population. Of the participants, 88% were females and 12% males (Table 1). No statistical difference in gender emerged regarding the vocal symptoms experienced. The distribution of the categories of schools and teachers is shown in Table 1. The frequency of experience of vocal problems before the introduction of distance teaching did not show significant differences between the categories.

**Table 1:** Background data (*n* = 121)

Teachers total		121
Gender	Female	106 (88%)
	Male	15 (12%)
Age groups	18–29 years	9 (7%)
	30–39 years	31 (26%)
	40–49 years	41 (34%)
	50–59 years	38 (31%)
	60–68 years	3 (2%)
Professional categories	Elementary school	43 (35%)
	Upper comprehensive school	43 (35%)
	Secondary school	35 (29%)
	Class teacher	41 (34%)
	Subject teacher	80 (66%)
Professional experience	Years (range)	1–38
	Years (mean)	15.45 (SD 9.6)
Chronic diseases	Chronic pulmonary disease	25 (21%)
	Allergic rhinitis	26 (21%)
	Reflux disease	8 (7%)
Operations		5 (4%)
Smoking	Never smoker	98 (81%)
	Ex-smoker or current smoker	23 (19%)

Of the teachers, 91% were aged 30–59 years (Table 1). The difference between the age groups experiencing vocal problems in regular teaching before the introduction of distance teaching was significant (*p* = 0.030). The 30–39 years age group experienced vocal problems more often than the other age groups (Table 2).

**Table 2:** Appearance of vocal problems during regular teaching (*n* = 121)

Vocal problems		No	Yes
Gender (*p* = 0.595)	Female	32 (28,8%)	79 (71,2%)
	Male	3 (30;0%)	7 (70,0%)
Age groups (*p* = 0.030)	18–29 years	5 (62,5%)	3 (37,5%)
	30–39 years	4 (13,3%)	26 (86,7%)
	40–49 years	11 (26,8%)	30 (73,2%)
	50–68 years	15 (35,7%)	27 (64,3%)
Chronic diseases (*p* = 0.002)	Chronic pulmonary disease	7 (28,0%)	18 (72,0)
	Allergic rhinitis	1 (3,7%)	26 (96,3%)
	Reflux disease	2 (28,6%)	5 (71,4%)
Operations (*p* = 0.329)	Not operated	33 (28,7%)	82 (71,3%)
	Operated	2 (33,3%)	4 (66,7%)
Smoking (*p* = 0.052)	Never smoker	32 (32,7%)	66 (67,3%)
	Ex-smoker or current smoker	3 (13,04%)	20 (86,96%)

Professional experience in the teaching profession varied from 1 to 38 years, with a mean of 15.45 years (SD 9.6) (Table 1). This variable was not associated with the frequency of vocal disorders in the classroom (*p* = 0.579).

Analysis of the whole group (Table 2) revealed that the number of previous surgical operations was not associated with experiencing vocal problems at school, but the frequency of illnesses such as chronic pulmonary diseases, reflux or especially allergic rhinitis was found to increase voice problems (*p* = 0.002).

Of the teachers, 81% were non-smokers (Table 1). Interestingly, smokers did not report significantly more vocal disorders (Table 2).

### Vocal problems before and during distance teaching

**Fig. 1:**
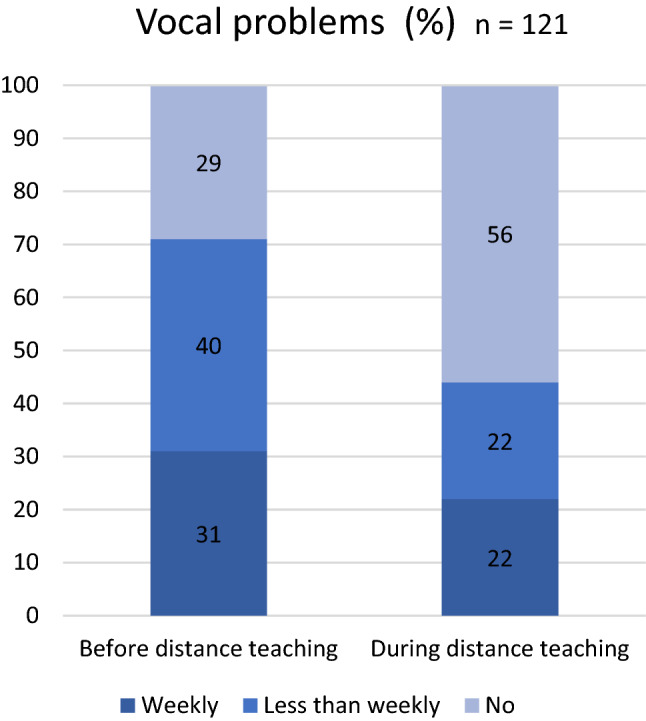
Frequency of vocal problems before and during distance teaching

Participants experienced significantly fewer voice disorders in distance teaching than in the preceding period (*p* < 0.001); which is shown in Fig. 1. Further, the mean value of VHI-10 (0 = no handicap, 40 = maximum handicap) decreased from 7.88 (SD 6.6) in school teaching to 4.58 (SD 6.1) in distance teaching (*p* < 0.001).

### Stress level, work ability and number of daily lessons

**Fig. 2:**
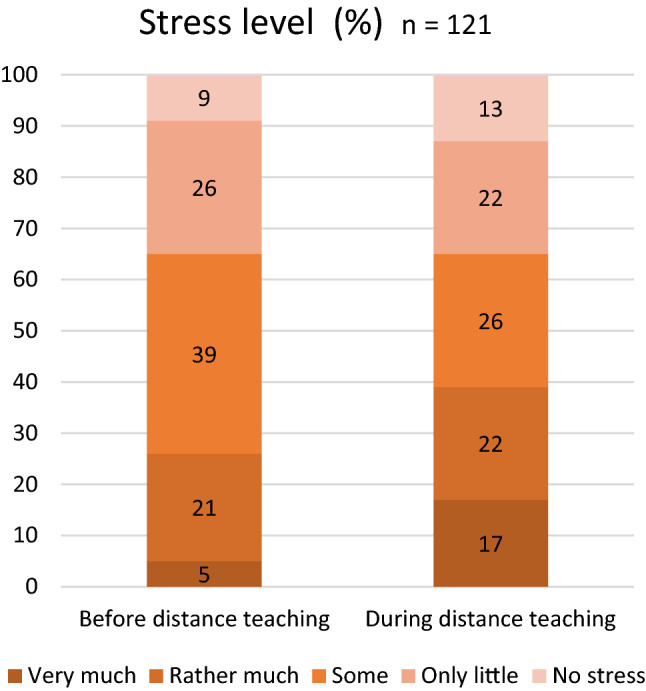
Stress level before and during distance teaching

In this study, the incidence of voice problems was associated with the stress level (from *p* = 0.002 to *p* = 0.034) and with the subjective ability to work (from *p* = 0.000 to *p* = 0.031). The distribution of the subjective stress level before and during distance teaching is shown in Fig. 2.

The mean value of the subjective ability to work (0 = not able to work, 10 = no inability) was before distance teaching 7.74 (SD 1.5) and during distance teaching 7.68 (SD 1.6). No significant difference emerged between the periods.

Of the teachers, 10% held seven or more (45-min) lessons, 59% five to six lessons, 22% three to four lessons and 9% zero to two lessons at school each day. By contrast, during distance teaching, 7% of teachers held seven or more lessons, 14% five to six lessons, 35% three to four lessons and 39% one to two lessons a day. The proportion of teachers holding no daily lessons during distance learning was 5%. However, the number of daily lessons did not influence the occurrence of voice-related problems.

### Acoustics, indoor quality, ergonomics and voice amplifiers

**Fig. 3:**
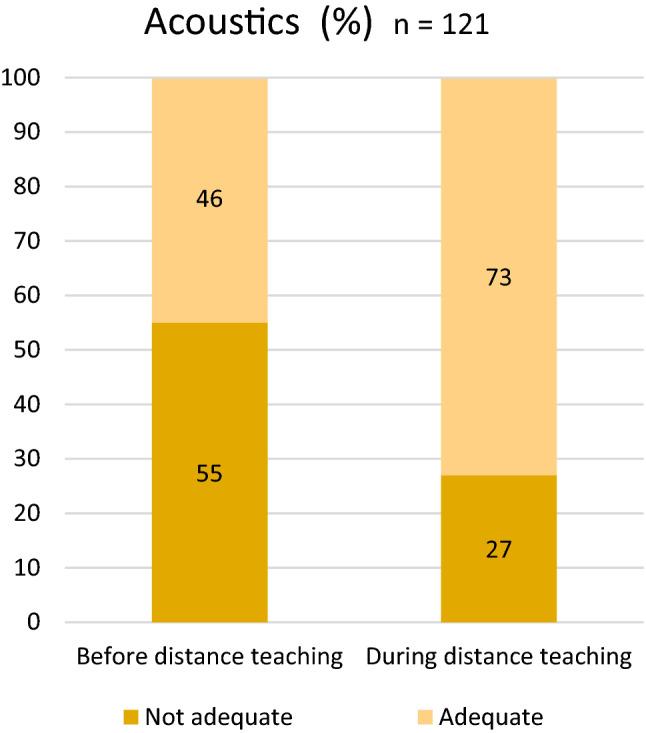
Acoustic conditions before and during distance teaching

Of the participants, 46% considered the acoustic conditions at the school (before distance teaching) to be adequate. During distance teaching 73% of teachers considered the acoustics at home to be adequate (Fig. 3).

Background noise was clearly identified as being an important factor causing voice-related problems during school and distance teaching. However, background noise caused subjective voice problems more often (*p* = 0.018/*p* = 0.002) and a higher VHI-10 score (*p* = 0.000/*p* = 0.000) in school classrooms than in distance teaching (Table 3).

**Table 3:** Subjectively experienced disturbing factors of the working environment (%) (*n* = 121)

	Not at all		Only little		Somehow		Rather much		Very much	
	Before distance teaching	During distance teaching	Before distance teaching	During distance teaching	Before distance teaching	During distance teaching	Before distance teaching	During distance teaching	Before distance teaching	During distance teaching
Background noise	6 (*n* = 7)	61 (*n* = 75)	15 (*n* = 17)	31 (*n* = 37)	31 (*n* = 38)	6 (*n* = 7)	31 (*n* = 38)	1 (*n* = 1)	17 (*n* = 21)	1 (*n* = 1)
Technical challenges	20 (25)	14 (*n* = 17)	35 (*n* = 42)	28 (*n* = 34)	36 (*n* = 44)	38 (*n* = 46)	7 (*n* = 8)	14 (*n* = 17)	2 (*n* = 2)	6 (*n* = 7)
Poor indoor quality	21 (*n* = 25)	86 (*n* = 105)	25 (*n* = 30)	8 (*n* = 9)	22 (*n* = 27)	3 (*n* = 4)	13 (*n*= 16)	2 (*n* = 2)	19 (*n* = 23)	1 (*n* = 1)
Poor ergonomics	22 (*n* = 27)	11 (*n* = 13)	28 (*n* = 34)	22 (*n* = 26)	30 (*n* = 36)	25 (*n* = 31)	16 (*n* = 19)	26 (*n* = 32)	4 (*n* = 5)	16 (*n* = 19)

Teachers reporting subjectively experienced poor indoor air quality at school had significantly more vocal problems (*p* = 0.003) and higher VHI-10 scores (*p* = 0.000) than teachers not reporting such a concern. During the distance teaching period, no such relationship appeared (Table 3).

Moreover, the ergonomic deficiencies at school had a significant influence on VHI-10 (*p* = 0.005), but not on vocal problems.

Loudspeakers were used in the classroom by 7% of teachers and 93% did not use any technical equipment to amplify their voice. During distance teaching, 46% of teachers used a headset, 8% used headphones, 4% a separate microphone, 2% a conference speaker and 41% used no technical acoustic equipment apart from the computer. Usage of different kinds of acoustic equipment at school or during distance teaching was not associated with the frequency of voice problems. The same was found in terms of the association of technical difficulties with the frequency of voice problems (Table 3).

## Discussion

The teaching profession is known to be associated with vocal disorders [2, 3]. Due to the COVID-19 pandemic, Finnish schools and educational institutions switched to distance learning for two months, to slow down the effects of the pandemic in Finland. Our aim here was to determine whether this change to distance teaching in the home working environment had an impact on teachers’ voice symptoms and work ability.

Results revealed that teachers experienced fewer vocal symptoms and had a lower VHI-10 score during distance teaching than during the regular teaching period at school. Accordingly, as the number of daily lessons did not influence the appearance of voice-related problems, distance teaching affected teachers’ voices in a positive manner. While the cause remains unknown, these significant differences might suggest distance teaching as a potential solution for teachers with vocal symptoms in certain situations. Nemr et al. showed in their questionnaire study (between July and October 2020) on Brazilian teachers working in various levels and fields of education that vocal self-perception improved during the pandemic compared with the pre-pandemic period [24].

In their questionnaire study, Roy et al. [25] demonstrated an increase in teachers´ voice disorders with age, peaking 50–59 years age group. Interestingly, in our survey the 30–39 years age group was associated with the highest frequency of vocal problems. The reason for this remains obscure. Furthermore, the cumulative number of years spent in the educational occupation had no significant influence on the occurrence of voice disorders.

Background noise was clearly shown to be an important factor causing voice-related problems in both periods and this was even more conspicuous in the classroom. It was associated positively with the frequency of subjective voice problems and the VHI-10 score, which is in line with previous studies [14]. Thus, these findings suggest that any realisations to improve acoustic conditions—in the classroom and in distance teaching—could reduce teachers´ vocal problems and enable them to withstand the effects of occupational voice use during the day better. The teachers considered acoustic conditions to be better in distance teaching. Thus, the forementioned improvements are especially needed in classrooms.

Another issue disclosed by this survey was the high amount of subjectively experienced poor indoor air quality among the teachers suffering from vocal problems. In addition, the ergonomic deficiencies at school (i.e., of electrically-adjustable desks) had an influence on teachers’ voices. These factors warrant attention in environmental arrangements in schools and require further investigations.

A positive effect of vocal amplifiers on teachers´ voices has been shown in earlier studies [26, 27]. In our investigation, neither the use of this kind of equipment nor related technical difficulties were associated with the frequency of voice disorders before or during distance teaching. It should be noted that we did not examine whether voice amplifiers helped teachers in school teaching relative to before the usage of voice amplifiers. Accordingly, this study does not imply that voice amplifiers are unhelpful.

The frequency of illnesses, such as chronic pulmonary diseases, reflux, and especially allergic rhinitis, was found to increase voice problems. This association confirms the findings from previous studies [28].

The stress level associated with more vocal symptoms, and vocal symptoms in turn were associated with a subjective weakened ability to work. However, both associations only describe an existing relationship, not its direction. In any case, these findings confirm previous research about the importance of taking care of teachers´ working conditions to avoid excessive sick leave.

Subjective stress was not significantly different between the two teaching periods. However, most of the teachers considered the workload to be higher in distance teaching. This might be the result of the unexpected and extensive modification of working methods without sufficient time to prepare. The workload can be reduced in the future if teachers are better prepared for distance teaching.

In contrast to what we expected, no significant difference emerged between subjective work ability before and during distance teaching.

### Study strengths and limitations

To our knowledge, this is the first study to examine the effects of distance teaching on voice problems among teachers in the first period of distance teaching during the COVID-19 pandemic. This study provides an insight into an unprecedented situation and how it affects one of the most important patient groups in voice clinics. In addition, participants covered all regions of Finland. Moreover, the questionnaire used an internationally accepted and validated instrument for measuring vocal health, namely the Vocal Health Index.

A limitation of the study was the exclusion of special education teachers, which was deliberate, to avoid the diversity of the sample.

By using e-identification we could exclude any participation by other persons or duplicate responses. On the other hand, due to the obligatory e-identification, teachers less interested in the subject might not have responded. Analogous to this, people not having any problems with their voice potentially participated less often.

The average completion time to fill in the survey was 3–5 min. We assume that it is long enough to cause dropouts. The approximative time was not mentioned at the beginning of the questionnaire, though.

Estimating the dropout rate was not possible due to the survey being an online one with advertisement done on social media platforms that not all teachers are members of and not necessarily online active when the survey was announced.

## Conclusions

This study provides an insight on the effect of distance teaching on teachers’ voices. We found fewer reports of voice problems among teachers in distance teaching than at school.

Acoustic conditions were reported as being more suitable in distance teaching, although many teachers used only their computer/smartphone without any more advanced acoustic equipment such as headsets. Background noise was the most disturbing factor for the teacher´s voice in the classroom, with its effect being somewhat ameliorated in distance teaching.

Subjectively experienced poor indoor air quality and ergonomic deficiencies at school were associated with teachers’ voice problems and warrant further investigation.

The plus-value resulting from the study suggests that distance teaching should be discussed as a potential solution for teachers suffering from voice problems at work, in addition to improving classroom acoustics and wearing a microphone.

## Supplementary Information

Below is the link to the electronic supplementary material.

Supplementary file1 (PDF 208 KB)

Supplementary file2 (PDF 264 KB)
