# Supplementary material for: Voice symptoms in teachers during distance teaching: a survey during the COVID-19 pandemic in Finland
Source: Eur Arch Otorhinolaryngol. 2021 Jul 4;278(11):4383–90. doi: 10.1007/s00405-021-06960-w (PMC8255054; doi:10.1007/s00405-021-06960-w)
Supplement: Supplementary file 1 — Supplementary file1 (PDF 208 KB) [file 405_2021_6960_MOESM1_ESM.pdf]

Opettajien äänikysely

1. Nimi

2. Sähköpostiosoite

3. Katuosoite *

4. Puhelinnumero

TAUSTATIEDOT

5. Ikä *

- 18 - 29 vuotta
- 30 - 39 vuotta
- 40 - 49 vuotta
- 50 - 59 vuotta
- 60 - 68 vuotta

6. Sukupuoli *

- Nainen

- ◯ Mies
- ◯ Muu

7. Onko sinulla todettu seuraavia sairauksia?

- ◯ Krooninen keuhkosairaus (esim. astma, COPD)
- ◯ Allerginen nuha
- ◯ Refluksitauti

8. Onko sinulle tehty nielun tai kurkunpään leikkausta?
Nielu- ja kitarisaleikkauksia ei huomioida. *

- ◯ Ei
- ◯ Kyllä
- ◯ En osaa sanoa

9. Jos vastasit kyllä, mikä leikkaus?

10. Tupakoitko?
(Tupakoinnilla tarkoitetaan keskimäärin yhden savukkeen polttamistä päivässä vähintään vuoden ajan.) *

- ◯ En ole koskaan tupakoinut
- ◯ Olen lopettanut tupakoinnin **ja** ollut yli vuoden tupakoimatta
- ◯ Tupakoin edelleen **tai** olen lopettanut alle vuosi sitten

11. Millä kouluasteella opetat pääasiassa? *

- ◯ Alakoulu

- Yläkoulu
- Lukio

12. Opetatko TÄLLÄ HETKELLÄ etä- vai lähiopetuksessa? *

- Etäopetuksessa
- Lähiopetuksessa
- Molemmissa

13. Ammatti *

- Luokanopettaja
- Aineenopettaja

14. Jatkokysymys aineenopettajille: Pääasiallinen opetettava aine?

- Äidinkieli ja kielet
- Reaaliaineet
- Matemaattiset aineet
- Taide- ja taitoaineet

15. Kuinka monta vuotta olet toiminut opettajana?
Laske kuluva vuosi yhdeksi vuodeksi. *

16. Missä maakunnassa työskentelet? *

- Ahvenanmaa
- Etelä-Karjala
- Etelä-Pohjanmaa
- Keski-Suomi
- Kymenlaakso
- Lappi
- Pohjois-Savo
- Päijät-Häme
- Satakunta

- ◯ Etelä-Savo
- ◯ Kainuu
- ◯ Kanta-Häme
- ◯ Keski-Pohjanmaa
- ◯ Pirkanmaa
- ◯ Pohjanmaa
- ◯ Pohjois-Karjala
- ◯ Pohjois-Pohjanmaa
- ◯ Uusimaa
- ◯ Varsinais-Suomi

Seuraavassa kysytään tilannetta ENNEN etäopetusjaksoa.

17. Kuinka monta oppituntia (45 min) keskimäärin puhuit työpäivän aikana ennen etäopetusjaksoa? *

- ◯ 0
- ◯ 1 - 2
- ◯ 3 - 4
- ◯ 5 - 6
- ◯ 7 tai enemmän

18. Oliko sinulla ääniongelmia ennen etäopetusjaksoa? *

- ◯ Ei
- ◯ Viikottain
- ◯ Harvemmin
- ◯ Ei lainkaan
- ◯ En osaa sanoa

Seuraavilla väittämillä monet ihmiset ovat kuvailleet omaa ääntään ja sen vaikutuksia elämäänsä. Valitse vastaus, joka kertoo kuinka usein sinusta tuntuu samalta.

Arvioi ääntäsi **ennen** etäopetusjaksoa.

19. Ääneni vuoksi ihmisten on vaikea kuulla minua. *

- Ei koskaan
- Ei juuri koskaan
- Joskus
- Melkein aina
- Aina

20. Ihmisten on vaikeaa ymmärtää mitä sanon meluisassa huoneessa. *

- Ei koskaan
- Ei juuri koskaan
- Joskus
- Melkein aina
- Aina

21. Ääniongelmat rajoittavat henkilökohtaista ja sosiaalista elämääni. *

- Ei koskaan
- Ei juuri koskaan
- Joskus
- Melkein aina
- Aina

22. Tunnen jääväni keskustelujen ulkopuolelle ääneni vuoksi. *

- Ei koskaan
- Ei juuri koskaan
- Joskus
- Melkein aina
- Aina

23. Ääniongelman vuoksi menetän tuloja. *

- Ei koskaan
- Ei juuri koskaan
- Joskus
- Melkein aina
- Aina

24. Minusta tuntuu, että joudun ponnistelemaan tuottaakseni ääntä. *

- Ei koskaan
- Ei juuri koskaan
- Joskus
- Melkein aina
- Aina

25. Ääneni selkeyttä on vaikea ennustaa. *

- Ei koskaan
- Ei juuri koskaan
- Joskus
- Melkein aina
- Aina

26. Ääniongelmani hermostuttaa minua. *

- Ei koskaan
- Ei juuri koskaan
- Joskus
- Melkein aina

Aina

27. Ääneni aiheuttaa minulle huomattavan haitan. *

- Ei koskaan
- Ei juuri koskaan
- Joskus
- Melkein aina
- Aina

28. Ihmiset kysyvät: "Mikä ääntäsi vaivaa?". *

- Ei koskaan
- Ei juuri koskaan
- Joskus
- Melkein aina
- Aina

29. Arvioi vielä, haittasivatko seuraavat asiat sinua ennen etäopetusjaksoa? *

	Ei lainkaan	Vain vähän	Jonkin verran	Melko paljon	Erittäin paljon
Melu					
Tekniset haasteet					
Huono sisäilma					
Huono työergonomia (esim. työpöytä)					

30. Koitko akustiset olosuhteet ennen etäopetusjaksoa (koulussa) työhösi sopiviksi? *

- Kyllä

- Ei
- En osaa sanoa

31. Käytitkö äänenvahvistinta ennen etäopetusjaksoa (koulussa)? *

- En käyttänyt
- Käytin

Stressillä tarkoitetaan tilannetta, jossa ihminen tuntee itsensä jännittyneeksi, levottomaksi, hermostuneeksi tai ahdistuneeksi taikka hänen on vaikea nukkua asioiden vaivatessa jatkuvasti mieltä.

32. Tunsitko ennen etäopetusjaksoa tällaista stressiä? *

- En lainkaan
- Vain vähän
- Jonkin verran
- Melko paljon
- Erittäin paljon

33. Oletetaan, että työkykysi on parhaimmillaan saanut 10 pistettä. Minkä pistemäärän antaisit työkyvyllesi ennen etätyöjaksoa? 0 tarkoittaa sitä, ettet olisi pystynyt lainkaan työhön. *

0	1	2	3	4	5	6	7	8	9	10
○	○	○	○	○	○	○	○	○	○	○

Seuraavassa kysytään tilannetta etäopetusjakson AIKANA.

34. Kuinka monta oppituntia (45 min) keskimäärin puhut/puhuit työpäivässä etäopetusjakson aikana? *

- 0
- 1 - 2

- 3 - 4
- 5 - 6
- 7 tai enemmän

35. Onko/oliko sinulla ääniongelmia etäopetusjakson aikana? *

- Ei
- Viikottain
- Harvemmin
- Ei lainkaan
- En osaa sanoa

36. Jos vastasit kyllä: Kuinka usein sinulla on/oli ääniongelmia?

- Päivittäin
- Viikottain
- Harvemmin

Seuraavilla väittämillä monet ihmiset ovat kuvailleet omaa ääntään ja sen vaikutuksia elämäänsä. Valitse vastaus, joka kertoo kuinka usein sinusta tuntuu samalta.

Arvioi ääntäsi etäopetuksen **aikana**.

37. Ääneni vuoksi ihmisten on vaikea kuulla minua. *

- Ei koskaan
- Ei juuri koskaan
- Joskus
- Melkein aina
- Aina

38. Ihmisten on vaikeaa ymmärtää mitä sanon meluisassa huoneessa. *

- Ei koskaan
- Ei juuri koskaan
- Joskus
- Melkein aina
- Aina

39. Ääniongelmat rajoittavat henkilökohtaista ja sosiaalista elämääni. *

- Ei koskaan
- Ei juuri koskaan
- Joskus
- Melkein aina
- Aina

40. Tunnen jääväni keskustelujen ulkopuolelle ääneni vuoksi. *

- Ei koskaan
- Ei juuri koskaan
- Joskus
- Melkein aina
- Aina

41. Ääniongelman vuoksi menetän tuloja. *

- Ei koskaan
- Ei juuri koskaan
- Joskus
- Melkein aina

- Aina

42. Minusta tuntuu, että joudun ponnistelemaan tuottaakseni ääntä. *

- Ei koskaan
- Ei juuri koskaan
- Joskus
- Melkein aina
- Aina

43. Ääneni selkeyttä on vaikea ennustaa. *

- Ei koskaan
- Ei juuri koskaan
- Joskus
- Melkein aina
- Aina

44. Ääniongelmani hermostuttaa minua. *

- Ei koskaan
- Ei juuri koskaan
- Joskus
- Melkein aina
- Aina

45. Ääneni aiheuttaa minulle huomattavan haitan. *

- Ei koskaan
- Ei juuri koskaan

◯ Joskus

◯ Melkein aina

◯ Aina

46. Ihmiset kysyvät: "Mikä ääntäsi vaivaa?". *

◯ Ei koskaan

◯ Ei juuri koskaan

◯ Joskus

◯ Melkein aina

◯ Aina

47. Arvioi vielä, haittaavatko/haittasivatko seuraavat asiat sinua etäopetusjakson aikana? *

	Ei lainkaan	Vain vähän	Jonkin verran	Melko paljon	Erittäin paljon
Melu	◯	◯	◯	◯	◯
Tekniset haasteet	◯	◯	◯	◯	◯
Huono sisäilma	◯	◯	◯	◯	◯
Huono työergonomia (esim. työpöytä yms.)	◯	◯	◯	◯	◯

48. Koitko akustiset olosuhteet etäopetuksen aikana työhösi sopiviksi? *

◯ Kyllä

◯ Ei

◯ En osaa sanoa

49. Käytätkö/käytitkö etäopetuksen aikana lisävarusteena *

◯ Korvakuulokkeita + mikrofonia (headset)

- Erillistä mikrofonia
- Erillisia korvakuulokkeita
- Konferenssikaiutinta
- En mitään edellä mainituista

50. Jos käytit headsetiä, minkä tyyppistä? (klikkaa kuvaa)

☐ 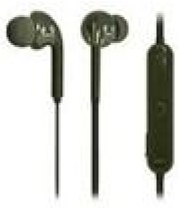

☐ 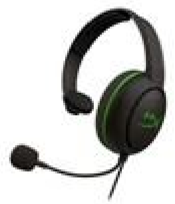

☐ 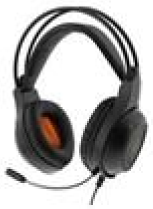

Stressillä tarkoitetaan tilannetta, jossa ihminen tuntee itsensä jännittyneeksi, levottomaksi, hermostuneeksi tai ahdistuneeksi taikka hänen on vaikea nukkua asioiden vaivatessa jatkuvasti mieltä.

51. Tunsitko etäopetusjakson aikana tällaista stressiä? *

- En lainkaan
- Vain vähän
- Jonkin verran
- Melko paljon
- Erittäin paljon

52. Oletetaan, että työkykysi on parhaimmillaan saanut 10 pistettä. Minkä pistemäärän antaisit työkyvyllesi etätyöjakson aikana? 0 tarkoittaa sitä, ettet pystyisi lainkaan työhön. *

0	1	2	3	4	5	6	7	8	9	10
○	○	○	○	○	○	○	○	○	○	○

53. Vertaa lopuksi työmäärääsi etä- ja lähiopetuksen välillä. *

- ◯ Lähiopetuksessa enemmän työtä
- ◯ Etäopetuksessa enemmän työtä
- ◯ Yhtä paljon työtä
- ◯ En osaa sanoa

Kiitos vastauksistasi!

Painamalla "Lähetä" ohjaudut suomi.fi-sivuston tunnistautumispalveluun.
