# Supplementary material for: Voice symptoms in teachers during distance teaching: a survey during the COVID-19 pandemic in Finland
Source: Eur Arch Otorhinolaryngol. 2021 Jul 4;278(11):4383–90. doi: 10.1007/s00405-021-06960-w (PMC8255054; doi:10.1007/s00405-021-06960-w)
Supplement: Supplementary file 2 — Supplementary file2 (PDF 264 KB) [file 405_2021_6960_MOESM2_ESM.pdf]

Teacher voice survey

1. Name

2. e-mail

3. Street address *

4. Telephone number

BACKGROUND DATA

5. Age *

- 18 - 29 years
- 30 - 39 years
- 40 - 49 years
- 50 - 59 years
- 60 - 68 years

6. Gender *

- Female

- ○ Male
- ○ Other

7. Have you been diagnosed with following diseases?

- ○ Chronic pulmonary disease (astma, COPD)
- ○ Allergic rhinitis
- ○ Reflux disease

8. Have you had previously pharyngeal or laryngeal surgery, except tonsillotomy or adenotomy? *

- ○ No
- ○ Yes
- ○ I don´t know

9. If you responsed yes, which operation?

10. Do you smoke?
(at least one cigarette a day for at least one year) *

- ○ I have never been smoking
- ○ I have ceased smoking **and** I have been non-smoker for at least one year
- ○ I am still smoking **or** I have been non-smoker less than one year ago

11. At what school level do you mainly teach? *

- ○ Primary school
- ○ Secondary school

◯ Senior high school

12. Are you CURRENTLY teaching in distance or regular teaching? *

◯ Distance teaching

◯ Regular teaching

◯ Both

13. Occupation *

◯ Class teacher

◯ Subject teacher

14. Follow-up question for subject teachers: Main subject to be taught?

◯ Finnish and languages

◯ Humanities and natural sciences

◯ Mathematical subjects

◯ Art and physical education

15. How many years have you been a teacher? Calculate the current year for one year. *

16. In which province do you work? *

◯ Ahvenanmaa

◯ Etelä-Karjala

◯ Etelä-Pohjanmaa

◯ Etelä-Savo

◯ Keski-Suomi

◯ Kymenlaakso

◯ Lappi

◯ Pirkanmaa

◯ Pohjois-Savo

◯ Päijät-Häme

◯ Satakunta

◯ Uusimaa

- Kainuu
- Kanta-Häme
- Keski-Pohjanmaa
- Pohjanmaa
- Pohjois-Karjala
- Pohjois-Pohjanmaa
- Varsinais-Suomi

The following will be asked about the situation BEFORE the distance learning session.

17. How many lessons (45 min) on average did you speak during the work day before distance learning period? *

- 0
- 1 - 2
- 3 - 4
- 5 - 6
- 7 or more

18. Did you experience any vocal problems before distance teaching? *

- No
- Weekly
- Rarely
- Not at all
- I do not know

These are statements that many people have used to describe their voices and the effects of their voices on their
lives. Choose the response that indicates how frequently you have the same experience.

Evaluate your voice how it was**before** the distance learning session.

19. My voice makes it difficult for people to hear me. *

- Never
- Almost never
- Sometimes
- Almost always
- Always

20. People have difficulty understanding me in a noisy room. *

- Never
- Almost never
- Sometimes
- Almost always
- Always

21. I am less outgoing because of my voice problem. *

- Never
- Almost never
- Sometimes
- Almost always
- Always

22. I feel left out of conversation because of my voice. *

- Never
- Almost never
- Sometimes
- Almost always
- Always

23. My voice problem causes me to lose income. *

- Never
- Almost never
- Sometimes
- Almost always
- Always

24. I feel as though I have to strain to produce voice. *

- Never
- Almost never
- Sometimes
- Almost always
- Always

25. The sound of my voice varies throughout the day. *

- Never
- Almost never
- Sometimes
- Almost always
- Always

26. My voice problem upsets me. *

- Never
- Almost never
- Sometimes
- Almost always

- Always

27. My voice makes me feel handicapped. *

- Never
- Almost never
- Sometimes
- Almost always
- Always

28. People ask, "What's wrong with your voice?". *

- Never
- Almost never
- Sometimes
- Almost always
- Always

29. Assess whether the following factors disturbed you at school? *

	Not at all	Only little	Somehow	Rather much	Very much
Noise	○	○	○	○	○
Technical challenges	○	○	○	○	○
Poor indoor air quality	○	○	○	○	○
Poor working ergonomics (e.g. worktop)	○	○	○	○	○

30. Did the acoustic conditions at school suit your work? *

- Yes
- No

◯ I do not know

31. Did you use a voice amplifier at school? *

◯ No

◯ Yes

Stress refers to a situation where a person feels tense, restless, nervous or anxious or having difficulty sleeping when things are constantly bothering the mind.

32. Did you experience this kind of stress at school? *

◯ Not at all

◯ Only little

◯ Some

◯ Rather much

◯ Very much

33. Assume that your ability to work has got 10 points at best. What score would you give your ability to work at school? 0 means you would not have been able to work at all. *

0	1	2	3	4	5	6	7	8	9	10
◯	◯	◯	◯	◯	◯	◯	◯	◯	◯	◯

The following will be asked about the situation DURING the distance learning session.

34. How many lessons (45 min) on average did you speak during the work day during distance learning period? *

◯ 0

◯ 1 - 2

- 3 - 4
- 5 - 6
- 7 or more

35. Did you experience any vocal problems during distance teaching? *

- No
- Weekly
- Rarely
- Not at all
- I do not know

36. If you responsed yes, how often did you experience vocal problems?

- Daily
- Weekly
- Rarely

These are statements that many people have used to describe their voices and the effects of their voices on their
lives. Choose the response that indicates how frequently you have the same experience.

Evaluate your voice how it was**during** the distance learning session.

37. My voice makes it difficult for people to hear me. *

- Never
- Almost never
- Sometimes
- Almost always
- Always

38. People have difficulty understanding me in a noisy room. *

- Never
- Almost never
- Sometimes
- Almost always
- Always

39. I am less outgoing because of my voice problem. *

- Never
- Almost never
- Sometimes
- Almost alwys
- Always

40. I feel left out of conversation because of my voice. *

- Never
- Almost never
- Sometimes
- Almost always
- Always

41. My voice problem causes me to lose income. *

- Never
- Almost never
- Sometimes
- Almost always

- Always

42. I feel as though I have to strain to produce voice. *

- Never
- Almost never
- Sometimes
- Almost always
- Always

43. The sound of my voice varies throughout the day. *

- Never
- Almost never
- Sometimes
- Almost always
- Always

44. My voice problem upsets me. *

- Never
- Almost never
- Sometimes
- Almost always
- Always

45. My voice makes me feel handicapped. *

- Never
- Almost never

- Sometimes
- Almost always
- Always

46. People ask, ‘‘What’s wrong with your voice?’’. *

- Never
- Almost never
- Sometimes
- Almost always
- Always

47. Assess whether the following factors disturbed you during distance learning session? *

	Not at all	Only little	Somehow	Rather much	Very much
Noise	◯	◯	◯	◯	◯
Technical challenges	◯	◯	◯	◯	◯
Poor indoor air quality	◯	◯	◯	◯	◯
Poor working ergonomics (e.g. worktop)	◯	◯	◯	◯	◯

48. Did the acoustic conditions during distance teaching suit your work? *

- Yes
- No
- I do not know

49. Did you use any of the following accessory during distance learning? *

- Earphones + microphone (headset)
- Separate microphone

- Separate earphones
- Office speakerphone
- None of above

50. If you used headset, which type? (click on the picture)

☐ 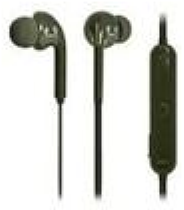

☐ 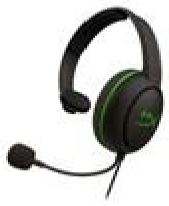

☐ 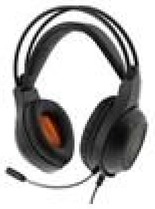

Stress refers to a situation where a person feels tense, restless, nervous or anxious or having difficulty sleeping when things are constantly bothering the mind.

51. Did you feel this kind of stress during distance teaching? *

- Not at all
- Only little
- Some
- Rather much
- Very much

52. Assume that your ability to work has got 10 points at best. What score would you give your ability to work during distance teaching? 0 means you would not have been able to work at all. *

0	1	2	3	4	5	6	7	8	9	10
○	○	○	○	○	○	○	○	○	○	○

53. Finally, compare your workload between distance and regular teaching *

- More work in regular teaching
- More work in distance teaching
- Just as much work in both
- I do not know

Thank you for your responses!

By pressing "Send", you are directed to the authentication service of the suomi.fi website.
